# Non-linear relationship of serum albumin-to-globulin ratio and cognitive function in American older people: a cross-sectional national health and nutrition examination survey 2011–2014 (NHANES) study

**DOI:** 10.3389/fpubh.2024.1375379

**Published:** 2024-04-26

**Authors:** Hang Yang, Zhenyi Liao, Ye Zhou, Zhenzhen Gao, Yajun Mao

**Affiliations:** Department of the Rehabilitation Medicine, The First Affiliated Hospital of Zhejiang Chinese Medical University, Hangzhou, China

**Keywords:** albumin-to-globulin ratio, cognition, aging, cross-sectional study, NHANES

## Abstract

**Background:**

Inflammation and liver function are associated with cognitive decline and dementia. Little is known about the serum albumin-to-globulin ratio on cognitive function.

**Objective:**

The objective of this study was to investigate the association between albumin-to-globulin ratio and cognitive function among the American older people.

**Methods:**

The public data available on the US National Health and Nutrition Examination Survey (NHANES) from 2011 to 2014 was used for this cross-sectional study. Participants aged ≥60 years completed the cognitive function assessments, including word learning and recall modules from the Consortium to Establish a Registry for Alzheimer’s Disease (CERAD), the animal fluency (AF) test, and the digit symbol substitution test (DSST). A composite cognition score was calculated to evaluate global cognition. The univariate and multivariate linear regression analysis, curve fitting, a threshold effect, along with a subgroup analysis and interaction tests were conducted.

**Results:**

Serum albumin-to-globulin ratio (per 0.1 unit) was positively associated DSST score (β = 0.36, 95% CI: 0.21, 0.51), AF score (β = 0.1, 95% CI: 0.04, 0.16) and global cognition score (β = 0.05, 95% CI: 0.02, 0.07), after being fully adjusted, while albumin-to-globulin ratio was not related to CERAD score (β = 0.05, 95% CI: −0.02, 0.12). A non-linear was observed in the dose–response relationship between albumin-to-globulin ratio and global cognition (*P* for non-linearity < 0.001). The subgroup analysis was overall stable, yet the interaction test was significant for age on global cognition (*P* for interaction = 0.036).

**Conclusion:**

The findings of this cross-sectional study suggested a positive and non-linear association between albumin-to-globulin ratio and cognitive function in the American older people. Maintaining albumin-to-globulin ratio with an appropriate range may be one of the therapeutic strategies to limit the progression of cognitive decline for the older people.

## Introduction

With the development of a hyper-aging society, the number of the older people with age-related cognitive decline increases, and the incidence of Alzheimer’s disease (AD) and Alzheimer’s disease-related dementia (ADRD) increases further, which almost doubles every 5 years after the age of 65 (1). The global prevalence of mild cognitive impairment (MCI) among individuals aged ≥65 is approximately 10–20% (2). MCI is characterized by a decline in cognition without significant impact on daily independent living, which represents a pre-stage on the continuum of cognitive decline between “normal aging” and dementia (3). Conservatively estimated, around 5–7% of individuals with MCI progress to dementia each year (4). While dementia progression is commonly viewed as irreversible, community-based studies suggested a reversion rate of approximately 25% for MCI (5). Dementia imposes a substantial burden on individuals, families, healthcare systems, and society at large. However, the therapeutic strategies for dementia are limited. Therefore, the proactive identification of pre-stage dementia and its associated factors, followed by primary interventions, is crucial in practice.

Research indicates that genetics, aging, nutrition, microbial exposure, infections, chronic inflammatory states, vascular risk, depression, and other factors are all associated with the etiology, development, and progression of cognitive impairment and dementia (6, 7). Current studies on cognitive impairment and dementia are focused on immunity and inflammation. Cells of the central nervous system are sensitive to peripheral inflammations and immune infiltration (8). The adaptive immunity decreases with aging, making older individuals more susceptible to infections and resulting in a chronic, low-degree inflammation, which is believed to be associated with cognition decline and dementia (9). Serum albumin is the most abundant protein in peripheral blood plasma and is associated with systemic inflammation. An elevated inflammatory index is an independent risk factor for hypoalbuminemia (10). The albumin could act as a chaperone to bind 90–95% of the β-amyloid protein (Aβ) in blood plasma, reducing the Aβ deposition in the brain (11). Shojai et al. reported an association between hypoalbuminemia and cognitive decline in the older people, as well as an adverse prognosis for other neurodegenerative diseases (12). The long-term chronic inflammation is often accompanied by an elevation in serum globulin levels, indicating excessive activity of the immune system, commonly observed in diseases such as chronic hepatitis, rheumatoid arthritis and cancer (13, 14). Both protein and globulin are synthesized by the liver, and many studies have increasingly focused on the significant role of liver in the development and progression of AD (15). The inflammatory response and immunization necessitate a comprehensive consideration of albumin and globulin levels, referred to as the albumin-to-globulin ratio (AGR) (16). There is emerging evidence that the lower AGR plays a significant role in the prognosis of cancers (16), heart failure (17), and infectious diseases (18).

At present, the evidence regarding the relationship between AGR and cognitive function is inadequate. Two studies conducted in Japan revealed a positive association between AGR and cognitive function (19, 20). The routine laboratory variables, albumin and globulin respectively, could predict cognitive decline through a machine learning algorithm study for those aged ≥75 years (21). Unfortunately, this study did not consider the ratio between the two. There is growing interest in identifying individuals with high risks of developing dementia. Therefore, more high-quality cohort studies are needed to explore the relationship between AGR and cognitive decline, AD, and ADRD, along with the underlying mechanisms.

In this study, we investigated the correlation between serum AGR and cognitive function among the American older people. In addition, we aimed to examine whether the association between AGR and cognition follows a linear pattern.

## Materials and methods

### Study population

NHANES began in the early 1960s and has been a major and continuous program conducted by the National Center for Health Statistics (NCHS). NHANES was designed in a stratified, multistage probability sampling survey to assess the health and nutritional statistics (22). Moreover, NHANES has a primary role in collecting extensive examinations for the US older people to increase the knowledge of the aging population. The National Center for Health Statistics (NCHS) Ethics Review Board subsequently approved the NHANES protocol (23). All participants provided written informed consent before participating. The public data and survey design are available at the NHANES website.^1^

This study used data from two NHANES cycles (2011–2012 and 2013–2014). SEQN stands for the respondent sequence number and is a unique identifier for each participant in NHANES, so that there are no duplicate subjects in these two cycles. A total of 2,765 participants with complete cognitive functioning assessment and AGR data were extracted from a data pool of 19,931 participants. The flowchart for participant enrollment is presented in (Supplementary Figure S1).

Serum albumin (g/dL) and globulin (g/dL) concentrations were measured by the DcX800 system as a biochromatic digital endpoint method. The albumin-to-globulin ratio (AGR) was calculated as the albumin divided by the globulin.

### Cognitive assessment

Participants aged ≥60 years are eligible for a series of cognitive assessments in NHANES (2011–2014), including word learning and recall modules from the Consortium to Establish a Registry for Alzheimer’s Disease (CERAD), the Animal Fluency test (AF) and the Digit Symbol Substitution test (DSST).

The CERAD Word Learning subtest, a major memory sub-domain to evaluate the immediate and delayed learning ability for new verbal information, includes three consecutive learning trials and a delayed recall. Every participant was instructed to read 10 unrelated words and was suggested to recall them immediately as many as possible for each trial. The delayed word recall occurred after completing AF and DSST. The maximum score for CERAD is 40.

The AF test was employed to evaluate the executive function by asking participants to name as many animals as possible in 1 min. The AF score is the total number of each named animal.

The DSST was a module from the Wechsler Adult Intelligence Scale (WAIS III) to assess processing speed, sustained attention, and working memory. Participants were asked to match the 133 corresponding symbols into the boxes next to the numbers in 2 min, with 133 being the highest score for DSST.

Additionally, a composite cognition score was created to represent global cognition and limit the uneven differences in individuals and the floor and ceiling effects (24), which was the summary of each standardized z-score of CERAD, AF, and DSST. The z-score was calculated as z = (x-m)/σ, where x shows the value of each individual, m presents the mean score of each test and σ is the standard deviation.

### Covariates

The possible potential confounding factors were assessed, including sociodemographic [age, gender, race and ethnicity, education level, marital status and poverty-to-income ratios (PIR)], lifestyle [smoking habit, drinking habit, physical activity, body mass index (BMI)], and comorbidities (history of hypertension, diabetes, coronary heart disease, stroke, and depression).

Ages were classified as 60–69 years, 70–79 years, and ≥80 years. Gender included male and female. Race and ethnicity included five groups: Mexican American, other Hispanic, non-Hispanic White, non-Hispanic Black, and other races. Education was classified as <9, 9–12, and >12 years. Marital status was divided into two groups: married or living with a partner and living alone. The PIR was classified as low (<1.3), medium (1.3–3.5) and high (≥3.5).

Lifestyle consisted of smoking habit (at least 100 cigarettes in life), alcohol drinking habit (at least 12 alcoholic drinks per year), and physical activity (at least 10 min moderate-intensity). BMI was divided into normal (<25 kg/m^2^), overweight (25–30 kg/m^2^) and obese (≥30 kg/m^2^).

The comorbidities of hypertension, diabetes, coronary heart disease, and stroke were diagnosed by self-reported physician diagnosis. Depression was defined as ≥10 scores according to the Patient Health Questionnaire (PHQ-9).

For covariates with missing data, such as PIR (missing 8.25%), drinking habit (missing 1.70%), PHQ-9 (missing 1.99%), BMI (missing 1.45%), coronary heart disease (missing 0.54%), hypertension (missing 0.18%), stroke (missing 0.1%), physical activity (missing 0.11%), diabetes (missing 0.07%), marital status (missing 0.07%), smoke habit (0.07%) and education level (missing 0.07%), we used multiple imputation, based on 5 replications and a chained equation approach method in the R mice procedure, as described in Van Buuren and Groothuis-Oudshoorn (25).

### Statistical analysis

No *a priori* calculation of statistical power was performed because the sample size was based on the available data from NHANES. All analyses were performed by R software (version 4.2.3; R Foundation for Statistical Computing)^2^ and Free Statistics software (version 1.9; Beijing Free Clinical Medical Technology Co., Ltd.). In all analyses, a two-sided *p*-value < 0.05 indicated statistical significance.

Firstly, the Kolmogorov–Smirnov test was used to determine the normality of continuous variables. Normally distributed variables were presented as mean (standard deviation), while skewed variables were presented as median (interquartile range, 25–75%). Categorical variables were represented by percentage (%). Statistical tests such as ANOVA, Kruskal–Wallis, and chi-squared tests were applied to compare differences across groups.

Then, the univariate and multivariate linear regression models were also used to examine the association of ARG and cognitive function in different dimensions. We converted AGR into a categorical variable according to the quartile and calculated the *P* for trend to verify the results of AGR as the continuous variable. Three models were adjusted based on clinical interest, previous scientific literature, or their associations with the outcomes of interest or a change in effect estimate of more than 10%. Model 1 was non-adjusted; Model 2 was adjusted for age, gender, race and ethnicity, education, marital status, and PIR; Model 3 was further adjusted for health habits (smoking, drinking, physical activity, and BMI) and comorbidities (hypertension, diabetes, coronary heart disease, stroke, depression).

We conducted a restricted cubic spline (RCS) with four knots (5th, 35th, 65th, and 95th) to explore the dose–response relationship between ARG and global cognition, modified by the cofounders consistent with model 3. We further developed a three-piecewise linear regression model of the relationship between AGR and global cognition. The subgroup analyses were also performed based on age, gender, race and ethnicity, PIR, BMI, and depression categories for interactions.

Finally, in the sensitivity analysis, we excluded individuals with missing covariate data and conducted multivariate linear regression and RCS to assess consistency with trends in individuals with complete data.

## Results

### Study population and baseline characteristics

A total number of 2,765 participants aged ≥60 years were included in the analysis. Table 1 shows the general characteristics of the participants according to AGR quartiles. Of those, the mean age was 69.5 (6.8) years, and 1,365 (49%) were male. Compared with other groups, group 4 (AGR ≥ 1.70) was more likely to be 60–69 years, male, Hispanic white, high education level, not living alone, and high level in PIR and a lower BMI. The higher AGR (≥1.70) was also associated with engagement in moderate physical activity, and a low incidence of diabetes and depression. In addition, participants had higher scores in all cognitive performance, including DSST, AF, CERAD, and global cognition. The basic characteristics of the excluded (AGR data missing) and included (AGR data completed) participants are shown in the Supplementary Table S1. Overall, the baseline data were stable, yet we cannot rule out the potential impact of non-random missing data on the results.

**Table 1:** Baseline characteristics of participants in NHANES, 2011–2014.

Characteristics	Participants, AGR quartiles					
	Total	Q1 (≤1.31)	Q2 (1.32–1.50)	Q3 (1.51–1.69)	Q4 (≥1.70)	*p*-value
No.	2,765	690	678	704	693	
Age, mean ± SD	69.5 ± 6.8	69.0 ± 6.7	69.3 ± 6.8	69.6 ± 6.8	70.0 ± 6.9	0.059
**Age, years**						0.448
60–69	1,498 (54.2)	393 (57)	368 (54.3)	383 (54.4)	354 (51.1)	
70–79	811 (29.3)	194 (28.1)	200 (29.5)	207 (29.4)	210 (30.3)	
≥80	456 (16.5)	103 (14.9)	110 (16.2)	114 (16.2)	129 (18.6)	
Gender (male), *n* (%)	1,356 (49.0)	324 (47)	324 (47.8)	356 (50.6)	352 (50.8)	0.371
**Race and ethnicity**						<0.001
Mexican American	244 (8.8)	63 (9.1)	63 (9.3)	71 (10.1)	47 (6.8)	
Other Hispanic	278 (10.1)	82 (11.9)	79 (11.7)	70 (9.9)	47 (6.8)	
Non-Hispanic White	1,351 (48.9)	188 (27.2)	299 (44.1)	393 (55.8)	471 (68)	
Non-Hispanic Black	627 (22.7)	286 (41.4)	172 (25.4)	104 (14.8)	65 (9.4)	
Others	265 (9.6)	71 (10.3)	65 (9.6)	66 (9.4)	63 (9.1)	
**Education level, years, *n* (%)**						<0.001
<9	310 (11.2)	104 (15.1)	88 (13)	69 (9.8)	49 (7.1)	
9–12	381 (13.8)	126 (18.3)	102 (15)	83 (11.8)	70 (10.1)	
>12	2074 (75.0)	460 (66.7)	488 (72)	552 (78.4)	574 (82.8)	
**Marital status**						<0.001
Married or living with a partner	1,604 (58.0)	353 (51.2)	385 (56.8)	412 (58.5)	454 (65.5)	
Living alone	1,161 (42.0)	337 (48.8)	293 (43.2)	292 (41.5)	239 (34.5)	
**PIR, *n* (%)**						<0.001
Low	824 (29.8)	254 (36.8)	224 (33)	197 (28)	149 (21.5)	
Medium	1,074 (38.8)	294 (42.6)	255 (37.6)	261 (37.1)	264 (38.1)	
High	867 (31.4)	142 (20.6)	199 (29.4)	246 (34.9)	280 (40.4)	
Smoking habits, *n* (%)	1,400 (50.6)	349 (50.6)	335 (49.4)	372 (52.8)	344 (49.6)	0.561
Drinking habits, *n* (%)	1894 (68.5)	430 (62.3)	448 (66.1)	497 (70.6)	519 (74.9)	<0.001
Moderate physical activity, *n* (%)	766 (27.7)	164 (23.8)	173 (25.5)	207 (29.4)	222 (32)	0.002
BMI, kg/m^2^, Mean ± SD	29.0 ± 6.3	30.8 ± 7.4	29.1 ± 6.4	28.5 ± 5.6	27.6 ± 5.2	<0.001
Hypertension, *n* (%)	1721 (62.2)	492 (71.3)	418 (61.7)	413 (58.7)	398 (57.4)	<0.001
**Diabetes, *n* (%)**						<0.001
Yes	633 (22.9)	211 (30.6)	150 (22.1)	142 (20.2)	130 (18.8)	
No	2007 (72.6)	451 (65.4)	491 (72.4)	535 (76)	530 (76.5)	
Bordline	125 (4.5)	28 (4.1)	37 (5.5)	27 (3.8)	33 (4.8)	
Coronary heart disease, *n* (%)	260 (9.4)	59 (8.6)	66 (9.7)	66 (9.4)	69 (10)	0.819
Stroke, *n* (%)	193 (7.0)	67 (9.7)	44 (6.5)	37 (5.3)	45 (6.5)	0.009
Depression, *n* (%)	252 (9.1)	91 (13.2)	62 (9.1)	60 (8.5)	39 (5.6)	<0.001
DSST score, mean ± SD	45.9 ± 17.3	40.4 ± 16.9	44.4 ± 16.9	47.5 ± 16.9	51.3 ± 16.6	<0.001
AF score, mean ± SD	16.6 ± 5.5	15.2 ± 5.1	16.1 ± 5.2	17.3 ± 5.4	17.9 ± 5.6	<0.001
CERAD score, mean ± SD	24.9 ± 6.5	24.2 ± 6.4	24.5 ± 6.6	25.1 ± 6.7	25.7 ± 6.3	<0.001
Global cognition, median (IQR)	0.0 (−1.7, 1.7)	−0.7 (−2.3, 0.8)	−0.2 (−2.0, 1.4)	0.3 (−1.5, 1.9)	0.7 (−0.9, 2.4)	<0.001

AGR, albumin-to-globulin ratio; BMI, body mass index; PIR, poverty to income ratio; DSST, Digit Symbol Substitution Test; AF, Animal Fluency; CERAD, Consortium to Establish a Registry for Alzheimer’s Disease; Q1-Q4, quartiles based on the median of albumin-to-globulin ratio; SD, standard deviation; IQR: Interquartile Range.

### The association between AGR and cognitive function

The univariate analysis indicated that age, race and ethnicity, education level, marital status, PIR, drinking, moderate physical activity, hypertension, diabetes, coronary heart disease, stroke and depression were correlated with cognitive function (Supplementary Table S2).

Table 2 presents the results of the multivariable linear regression analysis examining the association between AGR and DSST, AF, CERAD and global cognition. AGR (per 0.1 unit) was positively associated with DSST score (β = 0.36, 95% CI: 0.21, 0.51), AF score (β = 0.1, 95% CI: 0.04, 0.16) and global cognition (β = 0.05, 95% CI: 0.02, 0.07), after being fully adjusted. When comparing the individuals in the lowest quartile of AGR (Q1 ≤ 1.31), the fully adjusted effect β (95% CI) for DSST in Q4 (≥1.70) was 3.45 (95% CI: 2.04, 4.85); for AF, 1.1 (95% CI: 0.54, 1.65); for global cognition, 0.51 (95% CI: 0.3, 0.72). In addition, the results also indicated a similar trend for DSST, AF, and global cognition (*P* for trend < 0.001).

**Table 2:** Multivariable linear regression to assess the association of AGR with DSST, AF, CERAD, and global cognition.

Variable		β (95% CI)					
	No.	Model 1	*p*-value	Model 2	*p*-value	Model 3	*p*-value
**DSST socre**							
AGR^a^	2,765	1.1 (0.91, 1.29)	<0.001	0.41 (0.26, 0.56)	<0.001	0.36 (0.21, 0.51)	<0.001
**AGR quartiles**							
Q1 (≤1.31)	690	0 (Ref)		0 (Ref)		0 (Ref)	
Q2 (1.32–1.50)	678	4.03 (2.24, 5.81)	<0.001	1.32 (−0.03, 2.67)	0.055	0.91 (−0.42, 2.24)	0.179
Q3 (1.51–1.69)	704	7.13 (5.36, 8.90)	<0.001	2.2 (0.83, 3.57)	0.002	1.68 (0.32, 3.03)	0.015
Q4 (≥1.70)	693	10.95 (9.17, 12.72)	<0.001	4.05 (2.63, 5.46)	<0.001	3.45 (2.04, 4.85)	<0.001
*P* for trend	2,765		<0.001		<0.001		<0.001
**AF score**							
AGR^a^	2,765	0.27 (0.21, 0.33)	<0.001	0.11 (0.06, 0.17)	<0.001	0.1 (0.04, 0.16)	0.001
**AGR quartiles**							
Q1 (≤1.31)	690	0 (Ref)		0 (Ref)		0 (Ref)	
Q2 (1.32–1.50)	678	0.96 (0.39, 1.53)	0.001	0.32 (−0.20, 0.85)	0.226	0.25 (−0.28, 0.77)	0.354
Q3 (1.51–1.69)	704	2.18 (1.62, 2.74)	<0.001	1.03 (0.50, 1.57)	<0.001	0.93 (0.39, 1.46)	0.001
Q4 (≥1.70)	693	2.72 (2.15, 3.28)	<0.001	1.21 (0.66, 1.76)	<0.001	1.10 (0.54, 1.65)	<0.001
*P* for trend	2,765		<0.001		<0.001		<0.001
**CERAD score**							
AGR^a^	2,765	0.13 (0.06, 0.20)	0.001	0.05 (−0.02, 0.12)	0.175	0.05 (−0.02, 0.12)	0.182
**AGR quartiles**							
Q1 (≤1.31)	690	0 (Ref)		0 (Ref)		0 (Ref)	
Q2 (1.32–1.50)	678	0.3 (−0.38, 0.99)	0.387	0.01 (−0.62, 0.63)	0.985	−0.02 (−0.64, 0.61)	0.957
Q3 (1.51–1.69)	704	0.94 (0.26, 1.62)	0.007	0.39 (−0.24, 1.03)	0.224	0.34 (−0.30, 0.98)	0.3
Q4 (≥1.70)	693	1.55 (0.87. 2.24)	<0.001	0.72 (0.06, 1.37)	0.032	0.69 (0.03, 1.35)	0.041
*P* for trend	2,765		<0.001		0.016		0.023
**Global cognition**							
AGR^a^	2,765	0.13 (0.11, 0.16)	<0.001	0.05 (0.03, 0.07)	<0.001	0.05 (0.02, 0.07)	<0.001
**AGR quartiles**							
Q1 (≤1.31)	690	0 (Ref)		0 (Ref)		0 (Ref)	
Q2 (1.32–1.50)	678	0.45 (0.20, 0.71)	<0.001	0.14 (−0.06, 0.34)	0.184	0.1 (−0.10, 0.29)	0.349
Q3 (1.51–1.69)	704	0.95 (0.71, 1.20)	<0.001	0.38 (0.17, 0.58)	<0.001	0.32 (0.12, 0.52)	0.002
Q4 (≥1.70)	693	1.37 (1.12, 1.62)	<0.001	0.57 (0.35, 0.78)	<0.001	0.51 (0.30, 0.72)	<0.001
*P* for trend	2,765		<0.001		<0.001		<0.001

AGR, albumin-to-globulin ratio; BMI, body mass index; PIR, poverty to income ratio; DSST, Digit Symbol Substitution Test; AF, Animal Fluency; CERAD, Consortium to Establish a Registry for Alzheimer’s Disease; Q1-Q4, quartiles based on the median of albumin-to-globulin ratio; 95% CI, 95% Confidence Interval.

a AGR was entered as a continuous variable per change 0.1 unit.

Model 1: non-adjusted model.

Model 2: adjusted for age, gender, race, education, marital status, PIR.

Model 3: adjusted for model 2, additionally adjusted for BMI, drinking, smoking, physical activity, hypertension, diabetes, coronary heart disease, stroke, and depression.

However, AGR entered as a continuous variable was not related to the CERAD score (β = 0.05, 95% CI: −0.02, 0.12) in model 3. Interestingly, compared to the participants in Q1 (AGR ≤ 1.31), Q4 (AGR ≥ 1.70) (β = 0.69, 95% CI: 0.03, 1.35) had a higher score of CERAD in the fully adjusted model 3. The trend test also indicated a similar trend for CERAD (*P* for trend = 0.023).

### Analysis of the non-linear relationship between AGR and cognition

**Figure 1:**
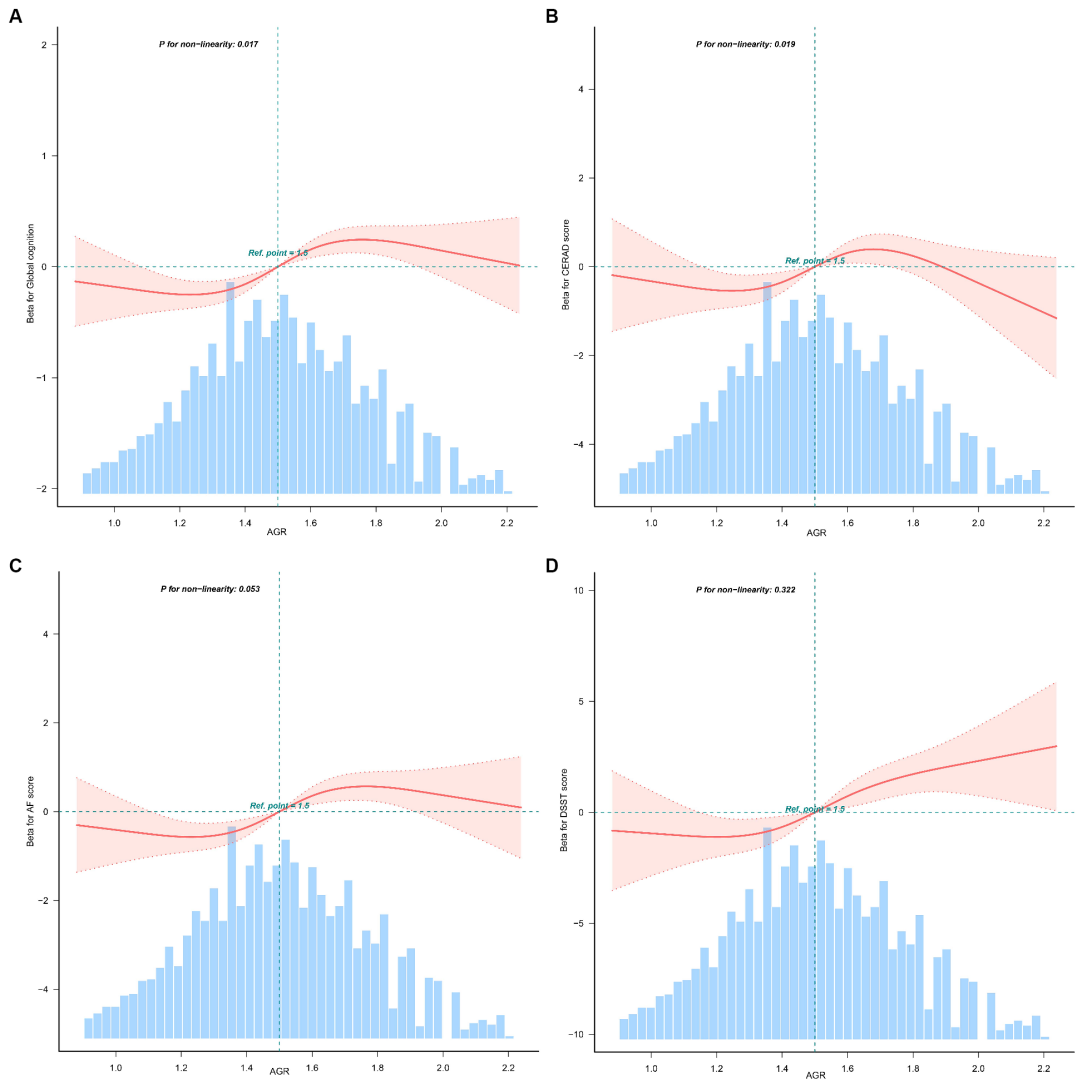
Non-linear relationship between AGR and global cognition, CERAD, AF, DSST (*n* = 2,765). AGR, albumin-to-globulin ratio; CERAD, Consortium to Establish a Registry for Alzheimer’s Disease; AF, Animal Fluency; DSST, Digit Symbol Substitution Test. **(A)** Relationship between AGR and global cognition; **(B)** relationship between AGR and CERAD; **(C)** relationship between AGR and AF; **(D)** relationship between AGR and DSST. Solid and dashed lines represent the predicted value and 95% confidence intervals. They were adjusted for age, gender, race, education, marital status, poverty-to-income ratio, body mass index, drinking, smoking, physical activity, hypertension, diabetes, coronary heart disease, stroke, and depression. Only 97.5% of the data is presented.

The relationship between AGR and global cognition was found to be nonlinear rather than linear (*P* for nonlinearity = 0.017, Figure 1A). Additionally, the association between AGR and CERAD was also observed (*P* for non-linearity = 0.019, Figure 1B). However, linear relationships were observed between AGR and AF (P for non-linearity = 0.053, Figure 1C), and DSST (P for non-linearity = 0.322, Figure 1D). The non-linearity relationship between AGR and global cognition indicated an S-shaped pattern. Using a three-piecewise linear regression model with all cofounders being adjusted, the global cognition score remained relatively flat but inverse until AGR was 1.22; subsequently, it started to increase by 0.1 (β = 0.10, 95% CI: 0.05, 0.16) for every 0.1-unit increase when AGR was in the range of 1.22 to 1.8; when AGR was beyond 1.81, the global cognition did not increase significantly (Table 3).

**Table 3:** Threshold analysis of AGR and global cognition.

	Global cognition	
	Adjusted model	
	β (95% CI)	*p*-value
**Three-piecewise linear regression model**		
AGR < 1.22^a^	0.05 (−0.15, 0.25)	0.635
AGR ≥ 1.22, <1.81^a^	0.10 (0.05, 0.16)	<0.001
AGR ≥ 1.81^a^	−0.17 (−0.35, 0.02)	0.823
Likelihood ratio test	–	0.019

AGR, albumin-to-globulin ratio; 95% CI, 95% Confidence Interval.

a AGR was entered as a continuous variable per change 0.1 unit.

Adjusted for age, gender, race, education, marital status, poverty-to-income ratio, body mass index, drinking, smoking, physical activity, hypertension, diabetes, coronary heart disease, stroke, and depression. Only 97.5% of the data is presented.

### Stratified and sensitivity analysis

**Figure 2:**
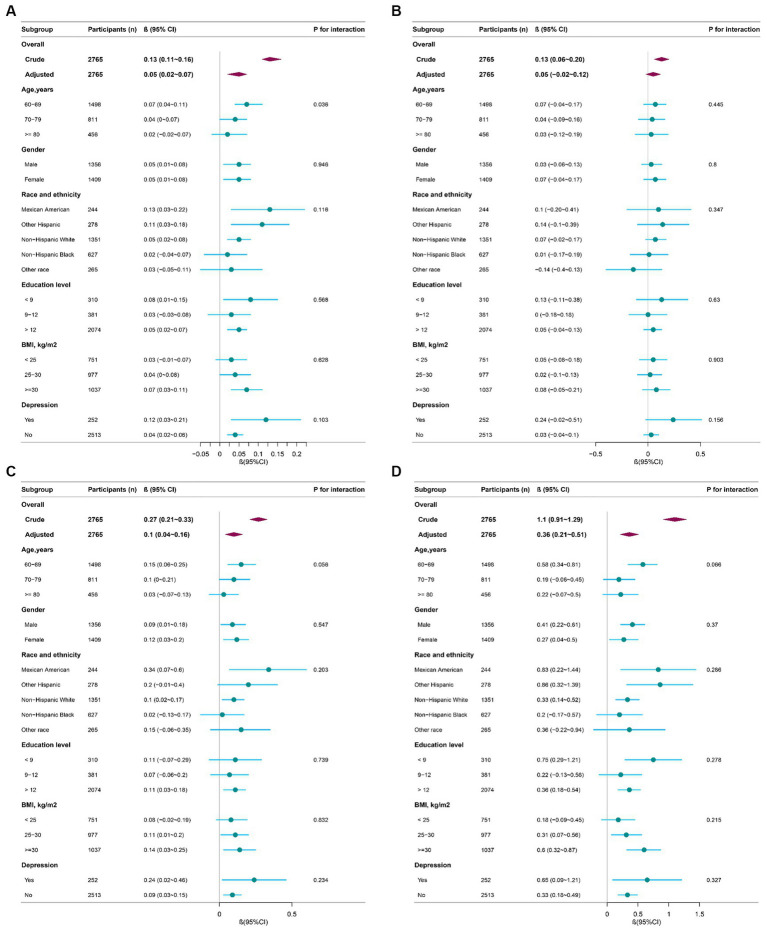
Effect size of AGR on global cognition, CERAD, AF, DSST in subgroups (*n* = 2,765). AGR, albumin-to-globulin ratio; CERAD, Consortium to Establish a Registry for Alzheimer’s Disease; AF, Animal Fluency; DSST, Digit Symbol Substitution Test. **(A)** Effect size of AGR on global cognition; **(B)** effect size of AGR on CERAD; **(C)** effect size of AGR on AF; **(D)** effect size of AGR on DSST. Adjusted for age, gender, race, education, marital status, poverty-to-income ratio, body mass index, drinking, smoking, physical activity, hypertension, diabetes, coronary heart disease, stroke, and depression.

We further performed stratified analyses of the relationship between AGR and global cognition, AF, CERAD and DSST (Figure 2). The effect (β) of global cognition, CERAD, AF, and DSST, in subgroups was stable overall. Yet, the interaction test was significant for age based on global cognition (*P* for interaction = 0.036).

During the sensitivity analysis, we compared differences between groups in the imputed variables (Supplementary Table S3), as the five datasets were generated after performing multiple imputations. The results indicated no statistically significant differences were found among the five datasets (*p* > 0.05). The results all suggested a non-linear relationship between AGR and Global cognition and CERAD (*P* for non-linearity < 0.05) for these five datasets. Similar results were observed when we conducted a multivariable linear regression and RCS (excluding the missing data of covariates, n = 2418) (Supplementary Table S4, Supplementary Figure S2).

## Discussion

This population-based cross-sectional study found a positive relationship between AGR and cognitive function among 2,765 American older people. Moreover, we identified a non-linear relationship between AGR and global cognition. The global cognition started to increase by 0.1 for every 0.1-unit increase in AGR when it reached 1.22 (*P* for nonlinearity < 0.001).

Our findings are in line with several recent studies. A large prospective cohort study conducted in China observed the ratio of AGR was significantly decreased in both the pathological and clinical progression of AD, in which AGR is significantly associated with cerebrospinal fluid β-amyloid_1–42_ (Aβ_1–42_) (*β* = 0.182, *p*_FDR_ < 0.001) (26). The positive association between AGR and cognitive function was observed in a case–control study and cross-sectional study in Japan, where the Mini-Mental State Examination (MMSE) and Montreal Cognitive Assessment (MoCA) were applied to assess cognitive function, respectively (19, 20). Chen et al. reported a higher AGR was associated with increased gray matter volume in the olfactory cortex and parahippocampal gyrus in young adults (27). Higher AGR means higher albumin and lower globulin (28). An elevated serum concentration of γ-globulin is an independent hematologic marker associated with cognitive decline in the dementia population (29). Therefore, it is reasonable to find out that AGR may serve as a protective factor and a potential indicator of cognitive performance.

A cross-sectional NHANES study detected a non-linear relationship between serum globulin and both AF and CERAD (30). The serum albumin presented a significant non-linear relationship with the risk of all-cause dementia (ACD) (31). Our study also detected a non-linear relationship between serum AGR and cognitive function which had never been reported before. Despite the clinical normal range of AGR being 1.0–2.0, there might be a possibility of cognitive decline associated with AGR even within this normal range. Therefore, we encourage maintaining AGR at an appropriate higher level. This is consistent with the finding of a prospective study conducted in Korea, indicating even within the normal range of serum albumin, individuals with albumin below 4.4 g/dL have an approximately three times higher risk of pathologic Aβ deposition (32). Our finding still needs further confirmation, but it indicated that poor nutrition or high inflammation in the older people may be linked to cognitive decline.

Additionally, our results showed a significant correlation between AGR and cognitive processing speed, sustained attention, and executive function (DSST, AF). However, there is no significant association with the immediate and delayed learning abilities related to the memory sub-domain (CERAD). This is largely consistent with several other cognitive studies based on the NHANES database (24, 30). A neuroimaging study identified an association between elevated AGR and the olfactory cortex and parahippocampal gyrus (27), which is involved in episodic memory and visuospatial processing (33). One explanation for this study may suggest the ability of execution and processing is more likely to be related to AGR. However, the underlying mechanism of AGR and cognitive dimensions needs further studies.

Increasing evidence suggests that inflammation is the leading culprit to dementia in the interaction between the down-regulated immune components, increased inflammation, and decreased neurogenesis (34). The amyloid cascade-inflammatory hypothesis has been proposed that Aβ peptide induces an inflammatory response that is enhanced by the presence of tau proteins, triggering the inflammatory activity of microglia, resulting in increased levels of inflammatory cytokines [tumor necrosis factor α, Interleukin 6, C-reactive protein (CRP)] (35). One meta-analysis investigated that chronic inflammatory diseases, elevated CRP, and other inflammatory factors increase vascular permeability, penetrate the blood–brain barrier, and infiltrate the brain, potentially developing dementia (36). The imbalance of AGR may reflect infection, malnutrition, chronic systematic inflammation, hepatic functional impairment, and autoimmunity (14, 37). AGR is related to cognition via reflection of homeostatic (non-high-density protein, high-CRP) (19). As the medium to long-term inflammatory, AGR was more sensitively related to the cognitive assessment than levels of CRP (20). Moreover, AGR has been demonstrated to be a valuable index for systemic inflammation in various diseases such as sepsis, systemic lupus erythematosus and rheumatoid arthritis (13, 38, 39).

New research is suggesting the role of the liver-brain axis for cognitive function as they might have potential communication pathways (27). Nho and colleagues highlighted the role of the liver in the involvement of metabolic disturbances in the progression of AD (40). The liver is the origin of brain Aβ deposits and is involved in the peripheral clearance of plasma Aβ through enzymes, albumin and lipid metabolism (15). There is a significant correlation between liver dysfunction and the volumes of the cognitive-related brain structures (hippocampus, thalamus, and amygdala regions) according to a prospective cohort study of 431,699 adults (31). The low AGR has been reported to be sensitively associated with liver malignancies (41). Besides the liver enzymes, albumin, globulin and the ratio of these two, play an active role in liver disease recovery (42).

There are some strengths of our study. The relationship between AGR and cognitive function was first investigated based on a national health and nutrition survey. Additionally, we considered a wide range of cofounders in this study. Further, a non-linear relationship between AGR and cognitive function was detected, suggesting a suitable range of AGR for better cognitive function. Although the mechanisms are not fully understood, our findings may have the potential to identify individuals at high risk of cognitive decline or dementia.

Our study also has limitations. First, all participants were Americans aged ≥60 years and the findings may not be generalizable to younger or populations of other races. Second, the cognitive assessments were DSST, AF, and CERAD, instead of the commonly used MMSE, MoCA, and the golden standard (European Consortium Criteria) for screening cognitive decline or mild cognitive impairment (43). Considering the reliability and validity of these cognitive assessments were compromised, we created a composite cognition z-score based on each test, assessing the global cognition domain (processing speed, sustained attention, working memory, executive function, and immediate and delayed memory). Moreover, our study was to investigate the relationship between AGR and cognitive performance, not MCI, AD, or ADRD. Thirdly, some residual confounders may exist, such as diet, medication interference, and other blood biomarkers. Finally, we could not make any causal inferences due to the cross-sectional study design. Future, prospective cohort studies are necessary to explore the cause-and-effect relationship between AGR and cognitive function.

## Conclusion

The findings of this cross-sectional study suggested a positive and non-linear association between AGR and cognitive function in American older people. Maintaining AGR with an appropriate range (we suggested over 1.22) may be one of the therapeutic strategies to limit the progression of cognitive decline for the older people.

## Data availability statement

The datasets presented in this study can be found in online repositories. The names of the repository/repositories and accession number(s) can be found below: http://www.cdc.gov/nchs/nhanes.htm.

## Ethics statement

The studies involving humans were approved by The National Center for Health Statistics (NCHS) Ethics Review Board. The studies were conducted in accordance with the local legislation and institutional requirements. The participants provided their written informed consent to participate in this study.

## Author contributions

HY: Conceptualization, Data curation, Formal analysis, Funding acquisition, Investigation, Methodology, Writing – original draft, Writing – review & editing. ZL: Conceptualization, Data curation, Investigation, Methodology, Writing – review & editing. YZ: Data curation, Investigation, Writing – original draft. ZG: Data curation, Formal analysis, Writing – review & editing. YM: Conceptualization, Supervision, Writing – review & editing, Writing – original draft.

## Footnotes

^1^ http://www.cdc.gov/nchs/nhanes.htm

^2^ http://www.Rproject.org
