# Supplementary material for: Non-linear relationship of serum albumin-to-globulin ratio and cognitive function in American older people: a cross-sectional national health and nutrition examination survey 2011–2014 (NHANES) study
Source: Front Public Health. 2024 Apr 26;12:1375379. doi: 10.3389/fpubh.2024.1375379 (PMC11082318; doi:10.3389/fpubh.2024.1375379)
Supplement: Supplementary file 1 [file Data_Sheet_1.docx]

**Supplementary Materials**

**Content**

**Supplementary Figure S1** Flow diagram of the screening and enrolment of study participants

**Supplementary Figure S2** Non-linear relationship of AGR and global cognition, CERAD, AF, DSST (n = 2418)

**Supplementary Table S1** The basic characteristics of the excluded (AGR data missing, n = 167) and included (AGR data completed, n = 2765) participants

**Supplementary Table S2** Univariate Linear Regressions Analysis (n = 2765)

**Supplementary Table S3** The differences in the covariates between the five imputation datasets (n = 2765)

**Supplementary Table S4** Multivariable linear regression to assess the association of AGR with DSST, AF, CERAD, and Global cognition (n = 2418)

**Supplementary Figures legends**

**Supplementary Figure S1** Flow diagram of the screening and enrolment of study participants

**Abbreviations:** NHANES, National Health and Nutrition Examination Survey

**Supplementary Figure S2** Non-linear relationship of AGR and global cognition, CERAD, AF, DSST (n = 2418)

**Abbreviations:** AGR, albumin-to-globulin ratio; CERAD, Consortium to Establish a Registry for Alzheimer’s Disease; AF, Animal Fluency; DSST, Digit Symbol Substitution Test

**Note:** (A), relationship between AGR and global cognition; (B), relationship between AGR and CERAD; (C), relationship between AGR and AF; (D), relationship between AGR and DSST

Solid and dashed lines represent the predicted value and 95% confidence intervals. They were adjusted for age, gender, race, education, marital status, poverty-to-income ratio, body mass index, drinking, smoking, physical activity, hypertension, diabetes, coronary heart disease, stroke, and depression. Only 97.5% of the data is presented.

**Supplementary Table S1** The basic characteristics of the excluded (AGR data missing, n = 167) and included (AGR data completed, n = 2765) participants.

| **Characteristics** | **Participants** | | |  |
| --- | --- | --- | --- | --- |
|  | **All**  **（n=2934）** | **AGR Missing**  **(n=169)** | **AGR Complete(n=2765)** | **P-value** |
| **Age, Mean ± SD** | 69.5 ± 6.8 | 69.6 ± 6.8 | 69.5 ± 6.8 | 0.818 |
| **Gender (Male), n (%)** | 1428 (48.7) | 72 (42.6) | 1356 (49) | 0.104 |
| **Race and ethnicity** |  |  |  | < 0.001 |
| Mexican American | 257 (8.8) | 13 (7.7) | 244 (8.8) |  |
| Other Hispanic | 296 (10.1) | 18 (10.7) | 278 (10.1) |  |
| Non-Hispanic White | 1401 (47.8) | 50 (29.6) | 1351 (48.9) |  |
| Non-Hispanic Black | 698 (23.8) | 71 (42) | 627 (22.7) |  |
| Others | 282 (9.6) | 17 (10.1) | 265 (9.6) |  |
| **Education level, years, n (%)** |  |  |  | 0.019 |
| < 9 | 330 (11.2) | 20 (11.8) | 310 (11.2) |  |
| 9-12 | 416 (14.2) | 36 (21.3) | 380 (13.7) |  |
| > 12 | 2188 (74.6) | 113 (66.9) | 2075 (75) |  |
| **Marital status** |  |  |  | 0.366 |
| Married or living with a partner | 1695 (57.8) | 92 (54.4) | 1603 (58) |  |
| Living alone | 1239 (42.2) | 77 (45.6) | 1162 (42) |  |
| **PIR, n (%)** |  |  |  | 0.014 |
| Low | 803 (29.9) | 56 (38.1) | 747 (29.4) |  |
| Medium | 1029 (38.3) | 53 (36.1) | 976 (38.5) |  |
| High | 852 (31.7) | 38 (25.9) | 814 (32.1) |  |
| **Smoking habits, n (%)** | 1488 (50.7) | 88 (52.1) | 1400 (50.6) | 0.717 |
| **Drinking habits, n (%)** | 1998 (68.1) | 101 (59.8) | 1897 (68.6) | 0.017 |
| **Moderate physical activity, n (%)** | 800 (27.3) | 34 (20.1) | 766 (27.7) | 0.032 |
| **BMI, kg/m^2^, Mean ± SD** | 29.0 ± 6.4 | 29.2 ± 7.1 | 29.0 ± 6.3 | 0.813 |
| **Hypertension, n (%)** | 1831 (62.4) | 111 (65.7) | 1720 (62.2) | 0.365 |
| **Diabetes, n (%)** |  |  |  | 0.017 |
| Yes | 689 (23.5) | 55 (32.5) | 634 (22.9) |  |
| No | 2113 (72.0) | 107 (63.3) | 2006 (72.5) |  |
| Borderline | 132 (4.5) | 7 (4.1) | 125 (4.5) |  |
| **Coronary heart disease, n (%)** | 270 (9.2) | 10 (5.9) | 260 (9.4) | 0.128 |
| **Stroke, n (%)** | 203 (6.9) | 11 (6.5) | 192 (6.9) | 0.829 |
| **Depression, n (%)** | 272 (9.3) | 15 (8.9) | 257 (9.3) | 0.855 |
| **DSST score, Mean ± SD** | 45.6 ± 17.3 | 40.9 ± 16.8 | 45.9 ± 17.3 | < 0.001 |
| **AF score, Mean ± SD** | 16.6 ± 5.5 | 15.4 ± 5.5 | 16.6 ± 5.5 | 0.005 |
| **CERAD score, Mean ± SD** | 24.8 ± 6.6 | 23.4 ± 7.0 | 24.9 ± 6.5 | 0.004 |
| **Global cognition score, Median (IQR)** | 0.0 (-1.7, 1.7) | -0.8 (-2.4, 1.0) | 0.1 (-1.6, 1.7) | < 0.001 |
| **AGR, Mean ± SD** | 1.5 ± 0.3 | 1.6 ± 0.3 | 1.5 ± 0.3 | < 0.001 |

**Notes:**

**Abbreviations:** AGR, albumin-to-globulin ratio; BMI, body mass index; PIR, poverty to income ratio; DSST, Digit Symbol Substitution Test; AF, Animal Fluency; CERAD, Consortium to Establish a Registry for Alzheimer’s Disease; SD, standard deviation; IQR: Interquartile Range

**Supplementary Table S2** Univariate Linear Regressions Analysis (n = 2765)

| **Variables** | **DSST score** | | **AF score** | | **CERAD score** | | | **Global cognition** |  |
| --- | --- | --- | --- | --- | --- | --- | --- | --- | --- |
|  | **β (95%CI)** | ***P*-value** | **β (95%CI)** | ***P*-value** | **β (95%CI)** | ***P*-value** | | **β (95%CI)** | ***P*-value** |
| **Age, years** | -0.71 (-0.8, -0.62) | < 0.001 | -0.17 (-0.20, -0.14) | < 0.001 | -0.28 (-0.31, -0.24) | < 0.001 | -0.11 (-0.13, -0.10) | | < 0.001 |
| **Age, years** |  |  |  |  |  |  |  | |  |
| 60-69 | 0(Ref) |  | 0(Ref) |  | 0(Ref) |  | 0(Ref) | |  |
| 70-79 | -5.77 (-7.23, -4.31) | < 0.001 | -1.31 (-1.78, -0.84) | < 0.001 | -1.86 (-2.41, -1.32) | < 0.001 | -0.86 (-1.06, -0.66) | | < 0.001 |
| ≥ 80 | -11.15 (-12.85, -9.46) | < 0.001 | -2.88 (-3.43, -2.34) | < 0.001 | -4.98 (-5.61, -4.35) | < 0.001 | -1.94 (-2.17, -1.70) | | < 0.001 |
| **Gender** |  |  |  |  |  |  |  | |  |
| Male | 0(Ref) |  | 0(Ref) |  | 0(Ref) |  | 0(Ref) | |  |
| Female | 4.94 (3.66, 6.22) | < 0.001 | -0.29 (-0.70, 0.12) | 0.161 | 2.4 (1.92, 2.88) | < 0.001 | 0.6 (0.42, 0.78) | | < 0.001 |
| **Race and ethnicity** |  |  |  |  |  |  |  | |  |
| Mexican American | 0(Ref) |  | 0(Ref) |  | 0(Ref) |  | 0(Ref) | |  |
| Other Hispanic | -3.07 (-5.87, -0.26) | 0.032 | -1.3 (-2.22, -0.38) | 0.005 | -0.9 (-2.02, 0.21) | 0.113 | -0.55 (-0.96, -0.15) | | 0.007 |
| Hispanic White | 10.83 (8.60, 13.06) | < 0.001 | 1.01 (0.29, 1.74) | 0.006 | 1.25 (0.36, 2.13) | 0.006 | 1.00 (0.68, 1.32) | | < 0.001 |
| Hispanic Black | -0.09 (-2.50, 2.33) | 0.945 | -1.68 (-2.46, -0.89) | < 0.001 | 0.65 (-0.31, 1.61) | 0.182 | -0.21 (-0.56, 0.13) | | 0.231 |
| Others | 10.38 (7.54, 13.22) | < 0.001 | -1.66 (-2.58, -0.73) | < 0.001 | 1.42 (0.29, 2.55) | 0.014 | 0.51 (0.11, 0.92) | | 0.013 |
| **Education level, years** |  |  |  |  |  |  |  | |  |
| < 9 | 0(Ref) |  | 0(Ref) |  | 0(Ref) |  | 0(Ref) | |  |
| 9-12 | 11.39 (9.13, 13.65) | < 0.001 | 0.6 (-0.19, 1.40) | 0.136 | 2.72 (1.78, 3.66) | < 0.001 | 1.19 (0.86, 1.51) | | < 0.001 |
| > 12 | 24.93 (23.14, 26.73) | < 0.001 | 3.56 (2.93, 4.19) | < 0.001 | 5.3 (4.55, 6.05) | < 0.001 | 2.9 (2.64, 3.17) | | < 0.001 |
| **Marital status** |  |  |  |  |  |  |  | |  |
| Married or living with a partner | 0(Ref) |  | 0(Ref) |  | 0(Ref) |  | 0(Ref) | |  |
| Living alone | -3.96 (-5.26, -2.66) | < 0.001 | -0.94 (-1.35, -0.53) | < 0.001 | -0.71 (-1.20, -0.22) | 0.005 | -0.51 (-0.69, -0.33) | | < 0.001 |
| **PIR** |  |  |  |  |  |  |  | |  |
| Low | 0(Ref) |  | 0(Ref) |  | 0(Ref) |  | 0(Ref) | |  |
| Medium | 7.26 (5.81, 8.71) | < 0.001 | 1.19 (0.71, 1.67) | < 0.001 | 1.67 (1.08, 2.25) | < 0.001 | 0.89 (0.69, 1.10) | | < 0.001 |
| High | 16.91 (15.38, 18.43) | < 0.001 | 3.27 (2.76, 3.78) | < 0.001 | 3.13 (2.52, 3.74) | < 0.001 | 2.06 (1.84, 2.27) | | < 0.001 |
| **Smoking habits** |  |  |  |  |  |  |  | |  |
| Yes | 0(Ref) |  | 0(Ref) |  | 0(Ref) |  | 0(Ref) | |  |
| No | 2.17 (0.88, 3.46) | < 0.001 | -0.01 (-0.41, 0.40) | 0.981 | 0.57 (0.08, 1.05) | 0.023 | 0.21 (0.03, 0.39) | | 0.021 |
| **Drinking habits** |  |  |  |  |  |  |  | |  |
| Yes | 0(Ref) |  | 0(Ref) |  | 0(Ref) |  | 0(Ref) | |  |
| No | -4.41 (-5.79, -3.03) | < 0.001 | -1.46 (-1.89, -1.02) | < 0.001 | -0.47 (-1.00, 0.05) | 0.076 | -0.59 (-0.79, -0.40) | | < 0.001 |
| **Moderate physical activity** |  |  |  |  |  |  |  | |  |
| Yes | 0(Ref) |  | 0(Ref) |  | 0(Ref) |  | 0(Ref) | |  |
| No | -4.75 (-6.18, -3.32) | < 0.001 | -1.72 (-2.17, -1.27) | < 0.001 | -1.16 (-1.70, -0.61) | < 0.001 | -0.77 (-0.97, -0.57) | | < 0.001 |
| **BMI** | 0 (-0.11, 0.10) | 0.943 | 0.03 (0.00, 0.06) | 0.07 | 0.06 (0.02, 0.10) | 0.002 | 0.01 (0.00, 0.03) | | 0.045 |
| **Hypertension** |  |  |  |  |  |  |  | |  |
| Yes | 0(Ref) |  | 0(Ref) |  | 0(Ref) |  | 0(Ref) | |  |
| No | 4.19 (2.86, 5.51) | < 0.001 | 1.33 (0.91, 1.75) | < 0.001 | 0.78 (0.27,1.28) | 0.002 | 0.6 (0.42, 0.79) | | < 0.001 |
| **Diabetes** |  |  |  |  |  |  |  | |  |
| Yes | 0(Ref) |  | 0(Ref) |  | 0(Ref) |  | 0(Ref) | |  |
| No | 6.8 (5.27, 8.32) | < 0.001 | 1.32 (0.83, 1.8) | < 0.001 | 1.12 (0.54, 1.70) | < 0.001 | 0.81 (0.59, 1.02) | | < 0.001 |
| Borderline | 5.58 (2.3, 8.86) | < 0.001 | 0.84 (-0.20, 1.89) | 0.113 | 0.34 (-0.91, 1.59) | 0.59 | 0.53 (0.07, 0.99) | | 0.024 |
| **Coronary heart disease** |  |  |  |  |  |  |  | |  |
| Yes | 0(Ref) |  | 0(Ref) |  | 0(Ref) |  | 0(Ref) | |  |
| No | 4.38 (2.18, 6.59) | < 0.001 | 0.6 (-0.10, 1.30) | 0.091 | 1.59 (0.76, 2.42) | < 0.001 | 0.61 (0.30, 0.91) | | < 0.001 |
| **Stroke** |  |  |  |  |  |  |  | |  |
| Yes | 0(Ref) |  | 0(Ref) |  | 0(Ref) |  | 0(Ref) | |  |
| No | 9.69 (7.18, 12.19) | < 0.001 | 2.09 (1.30, 2.89) | < 0.001 | 2.52 (1.57, 3.47) | < 0.001 | 1.33 (0.98, 1.68) | | < 0.001 |
| **Depression** |  |  |  |  |  |  |  | |  |
| Yes | 0(Ref) |  | 0(Ref) |  | 0(Ref) |  | 0(Ref) | |  |
| No | -9.1 (-11.31, -6.88) | < 0.001 | -1.99 (-2.69, -1.29) | < 0.001 | -1.17 (-2.01, -0.33) | 0.007 | -1.07 (-1.38, -0.76) | | < 0.001 |
| **AGR (per 0.1 unit)** | 1.1 (0.91, 1.29) | < 0.001 | 0.27 (0.21, 0.33) | < 0.001 | 0.13 (0.06, 0.20) | < 0.001 | 0.13 (0.11, 0.16) | | < 0.001 |

**Notes:**

**Abbreviations:** AGR, albumin-to-globulin ratio; BMI, body mass index; PIR, poverty to income ratio; DSST, Digit Symbol Substitution Test; AF, Animal Fluency; CERAD, Consortium to Establish a Registry for Alzheimer’s Disease; Q1-Q4, quartiles based on the median of albumin-to-globulin ratio; SD, standard deviation; IQR: Interquartile Range

**Supplementary Table S3** The differences in the covariates between the five imputation datasets

| **Variables** | **Total** | **IMP 1** | **IMP 2** | **IMP 3** | **IMP 4** | **IMP 5** | **P-value** | **statistic** |
| --- | --- | --- | --- | --- | --- | --- | --- | --- |
| **No.** | 13825 | 2765 | 2765 | 2765 | 2765 | 2765 |  |  |
| **Education level,years, n (%)** |  |  |  |  |  |  | 1 | 0.008 |
| < 9 | 1553 (11.2) | 310 (11.2) | 310 (11.2) | 311 (11.2) | 311 (11.2) | 311 (11.2) |  |  |
| 9-12 | 1902 (13.8) | 380 (13.7) | 381 (13.8) | 381 (13.8) | 380 (13.7) | 380 (13.7) |  |  |
| > 12 | 10370 (75.0) | 2075 (75) | 2074 (75) | 2073 (75) | 2074 (75) | 2074 (75) |  |  |
| **Marital status** |  |  |  |  |  |  | 1 | 0 |
| Married or living with a partner | 8020 (58.0) | 1604 (58) | 1604 (58) | 1604 (58) | 1604 (58) | 1604 (58) |  |  |
| Living alone | 5805 (42.0) | 1161 (42) | 1161 (42) | 1161 (42) | 1161 (42) | 1161 (42) |  |  |
| **PIR, Median (IQR)** | 2.2 (1.2, 4.2) | 2.1 (1.2, 4.2) | 2.1 (1.2, 4.1) | 2.2 (1.2, 4.2) | 2.2 (1.2, 4.2) | 2.2 (1.2, 4.2) | 0.981 | 0.415 |
| **PIR, n (%)** |  |  |  |  |  |  | 1 | 0.588 |
| Low | 4093 (29.6) | 822 (29.7) | 824 (29.8) | 817 (29.5) | 814 (29.4) | 816 (29.5) |  |  |
| Medium | 5321 (38.5) | 1063 (38.4) | 1074 (38.8) | 1060 (38.3) | 1062 (38.4) | 1062 (38.4) |  |  |
| High | 4411 (31.9) | 880 (31.8) | 867 (31.4) | 888 (32.1) | 889 (32.2) | 887 (32.1) |  |  |
| **BMI, kg/m^2^, Mean ± SD** | 29.0 ± 6.3 | 29.0 ± 6.3 | 29.0 ± 6.3 | 29.0 ± 6.3 | 29.1 ± 6.3 | 29.1 ± 6.3 | 1 | 0.011 |
| **Smoking habits, n (%)** | 6999 (50.6) | 1399 (50.6) | 1400 (50.6) | 1400 (50.6) | 1400 (50.6) | 1400 (50.6) | 1 | 0.001 |
| **Drinking habits, n (%)** | 9477 (68.5) | 1900 (68.7) | 1894 (68.5) | 1895 (68.5) | 1893 (68.5) | 1895 (68.5) | 1 | 0.049 |
| **Moderate physical activity, n (%)** | 3830 (27.7) | 766 (27.7) | 766 (27.7) | 766 (27.7) | 766 (27.7) | 766 (27.7) | 1 | 0 |
| **Hypertension, n (%)** | 8611 (62.3) | 1723 (62.3) | 1721 (62.2) | 1723 (62.3) | 1722 (62.3) | 1722 (62.3) | 1 | 0.004 |
| **Diabetes, n (%)** |  |  |  |  |  |  | 1 | 0.002 |
| Yes | 3166 (22.9) | 633 (22.9) | 633 (22.9) | 634 (22.9) | 633 (22.9) | 633 (22.9) |  |  |
| No | 10034 (72.6) | 2007 (72.6) | 2007 (72.6) | 2006 (72.5) | 2007 (72.6) | 2007 (72.6) |  |  |
| Borderline | 625 (4.5) | 125 (4.5) | 125 (4.5) | 125 (4.5) | 125 (4.5) | 125 (4.5) |  |  |
| **Coronary heart disease, n (%)** | 1295 (9.4) | 258 (9.3) | 260 (9.4) | 259 (9.4) | 259 (9.4) | 259 (9.4) | 1 | 0.009 |
| **Stroke, n (%)** | 962 (7.0) | 192 (6.9) | 193 (7) | 192 (6.9) | 192 (6.9) | 193 (7) | 1 | 0.007 |
| **Depression, n (%)** | 1272 (9.2) | 252 (9.1) | 252 (9.1) | 255 (9.2) | 257 (9.3) | 256 (9.3) | 0.999 | 0.092 |

**Notes:**

**Abbreviations:** IMP, imputation; PIR, poverty to income ratio; BMI, body mass index; SD, standard deviation; IQR: Interquartile Range

**Supplementary Table S4** Multivariable linear regression to assess the association of AGR with DSST, AF, CERAD, and Global cognition (n = 2418 ^b^)

|  |  | **β （95% CI）** | | | | | |
| --- | --- | --- | --- | --- | --- | --- | --- |
| **Variable** | **No.** | **Model 1** | **P-value** | **Model 2** | **P-value** | **Model 3** | **P-value** |
| **DSST socre** |  |  |  |  |  |  |  |
| **AGR^a^** | 2418 | 1.05 (0.85, 1.25) | <0.001 | 0.38 (0.22, 0.54) | <0.001 | 0.33 (0.17, 0.49) | <0.001 |
| **AGR quartiles** |  |  |  |  |  |  |  |
| Q1 (≤1.31) | 602 | 0(Ref) |  | 0(Ref) |  | 0(Ref) |  |
| Q2 (1.32-1.50) | 585 | 4.3 (2.41, 6.19) | <0.001 | 1.09 (-0.34, 2.52) | 0.136 | 0.71 (-0.70, 2.13) | 0.322 |
| Q3 (1.51-1.69) | 631 | 7.11 (5.26, 8.97) | <0.001 | 2.07 (0.63, 3.52) | 0.005 | 1.61 (0.18, 3.04) | 0.028 |
| Q4 (≥1.70) | 600 | 10.85 (8.97,12.73) | <0.001 | 3.68 (2.18, 5.18) | <0.001 | 3.20 (1.70, 4.70) | <0.001 |
| *P* for trend | 2418 |  | <0.001 |  | <0.001 |  | <0.001 |
|  |  |  |  |  |  |  |  |
| **AF score** |  |  |  |  |  |  |  |
| **AGR^a^** | 2418 | 0.26 (0.20, 0.33) | <0.001 | 0.11 (0.04, 0.17) | 0.001 | 0.1 (0.03~0.16) | 0.003 |
| **AGR quartiles** |  |  |  |  |  |  |  |
| Q1 (≤1.31) | 602 | 0(Ref) |  | 0(Ref) |  | 0(Ref) |  |
| Q2 (1.32-1.50) | 585 | 1.12 (0.51, 1.73) | <0.001 | 0.41 (-0.16, 0.97) | 0.159 | 0.34 (-0.23, 0.90) | 0.242 |
| Q3 (1.51-1.69) | 631 | 2.13 (1.53, 2.73) | <0.001 | 0.92 (0.35, 1.50) | 0.002 | 0.82 (0.25, 1.39) | 0.005 |
| Q4 (≥1.70) | 600 | 2.73 (2.12, 3.34) | <0.001 | 1.14 (0.55, 1.74) | <0.001 | 1.04 (0.44, 1.64) | 0.001 |
| *P* for trend | 2418 |  | <0.001 |  | <0.001 |  | <0.001 |
|  |  |  |  |  |  |  |  |
| **CERAD score** |  |  |  |  |  |  |  |
| **AGR^a^** | 2418 | 0.13 (0.06, 0.21) | 0.001 | 0.05 (-0.02, 0.13) | 0.148 | 0.05 (-0.02, 0.12) | 0.189 |
| **AGR quartiles** |  |  |  |  |  |  |  |
| Q1 (≤1.31) | 602 | 0(Ref) |  | 0(Ref) |  | 0(Ref) |  |
| Q2 (1.32-1.50) | 585 | 0.60 (-0.13, 1.34) | 0.107 | 0.19 (-0.48, 0.86) | 0.572 | 0.14 (-0.53, 0.82) | 0.675 |
| Q3 (1.51-1.69) | 631 | 1.06 (0.34, 1.78) | 0.004 | 0.52 (-0.16, 1.20) | 0.132 | 0.43 (-0.25, 1.11) | 0.213 |
| Q4 (≥1.70) | 600 | 1.66 (0.93, 2.39) | <0.001 | 0.77 (0.07, 1.47) | 0.031 | 0.73 (0.02, 1.44) | 0.044 |
| *P* for trend | 2418 |  | <0.001 |  | 0.02 |  | 0.031 |
|  |  |  |  |  |  |  |  |
| **Global cognition** |  |  |  |  |  |  |  |
| **AGR^a^** | 2418 | 0.13 (0.1~0.16) | <0.001 | 0.05 (0.03, 0.07) | <0.001 | 0.04 (0.02, 0.07) | <0.001 |
| **AGR quartiles** |  |  |  |  |  |  |  |
| Q1 (≤1.31) | 602 | 0(Ref) |  | 0(Ref) |  | 0(Ref) |  |
| Q2 (1.32-1.50) | 585 | 0.55 (0.28~0.82) | <0.001 | 0.17 (-0.05, 0.38) | 0.128 | 0.13 (-0.09, 0.34) | 0.25 |
| Q3 (1.51-1.69) | 631 | 0.97 (0.71~1.23) | <0.001 | 0.37 (0.15, 0.59) | 0.001 | 0.31 (0.09, 0.53) | 0.005 |
| Q4 (≥1.70) | 600 | 1.39 (1.12~1.66) | <0.001 | 0.54 (0.32, 0.77) | <0.001 | 0.49 (0.26, 0.72) | <0.001 |
| *P* for trend | 2418 |  | <0.001 |  | <0.001 |  | <0.001 |

**Notes:**

**Abbreviations:** AGR, albumin-to-globulin ratio; BMI, body mass index; PIR, poverty to income ratio; DSST, Digit Symbol Substitution Test; AF, Animal Fluency; CERAD, Consortium to Establish a Registry for Alzheimer’s Disease; Q1-Q4, quartiles based on the median of albumin-to-globulin ratio; 95% CI, 95% Confidence Interval

^a^AGR was entered as a continuous variable per change 0.1 unit.

^b^2418 was the number of participants with complete all covariates data.

Model 1: non-adjusted model

Model 2: adjusted for age, gender, race, education, marital status, PIR

Model 3: adjusted for model 2, additionally adjusted for BMI, drinking, smoking, physical activity, hypertension, diabetes, coronary heart disease, stroke, and depression
